# Constrained posture in dentistry – a kinematic analysis of dentists

**DOI:** 10.1186/s12891-017-1650-x

**Published:** 2017-07-05

**Authors:** Daniela Ohlendorf, Christina Erbe, Jennifer Nowak, Imke Hauck, Ingo Hermanns, Dirk Ditchen, Rolf Ellegast, David A. Groneberg

**Affiliations:** 1 Institute of Occupational Medicine, Social Medicine and Environmental Medicine, Goethe-University, Theodor-Stern-Kai 7, 60590 Frankfurt am Main, Germany; 2 School of Dentistry, Department of Orthodontics, University Medical Centre of the Johannes Gutenberg-University Mainz, Augustusplatz 2, 55131 Main, Germany; 3 Institute for Occupational Health and Safety (IFA) of the German Social Accident Insurance (DGUV), Alte Heerstraße 111, 53757 Sankt Augustin, Germany

**Keywords:** Cuela, Kinematic analysis, Dentist, Musculoskeletal disorder, Constrained posture

## Abstract

**Background:**

How a dentist works, such as the patterns of movements performed daily, is also largely affected by the workstation Dental tasks are often executed in awkward body positions, thereby causing a very high degree of strain on the corresponding muscles. The objective of this study is to detect those dental tasks, during which awkward postures occur most frequently. The isolated analysis of static postures will examine the duration for which these postures are maintained during the corresponding dental, respectively non-dental, activities.

Methods: 21 (11f/10 m) dentists (age: 40.1 ± 10.4 years) participated in this study. An average dental workday was collected for every subject. To collect kinematic data of all activities, the CUELA system was used. Parallel to the kinematic examination, a detailed computer-based task analysis was conducted. Afterwards, both data sets were synchronized based on the chronological order of the postures assumed in the trunk and the head region. All tasks performed were assigned to the categories “treatment” (I), “office” (II) and “other activities” (III). The angle values of each body region (evaluation parameter) were examined and assessed corresponding to ergonomic standards. Moreover, this study placed a particular focus on static positions, which are held statically for 4 s and longer.

**Results:**

For “treatment” (I), the entire head and trunk area is anteriorly tilted while the back is twisted to the right, in (II) and (III) the back is anteriorly tilted and twisted to the right (non-neutral position). Static positions in (I) last for 4–10s, static postures (approx. 60%) can be observed while in (II) and (III) in the back area static positions for more than 30 s are most common. Moreover, in (II) the back is twisted to the right for more than 60 s in 26.8%.

**Conclusion:**

Awkward positions are a major part of a dentists’ work. This mainly pertains to static positions of the trunk and head in contrast to “office work.” These insights facilitate the quantitative description of the dentist profession with regard to the related physical load along with the health hazards to the musculoskeletal system. Moreover, the results allow for a selective extraction of the most unfavorable static body positions that dentists assume for each of the activities performed.

## Background

In recent years, ergonomics applied in dentistry has gained increasing relevance. This is mainly due to the constantly rising numbers of dentists, who have expressed work-related pain pathologies, especially in the in the neck, shoulder and/or back area [1–14]. One of the main causes of the emergence of muscular imbalances and, accordingly, muscular problems is the unsuitable posture dentists take during work [5, 15]. Alghadir et al. [16] conducted a survey which reveals that there is a high prevalence of work-related musculoskeletal disorders (MSD) among dental professionals (85% of the respondents) after they started their profession. Factors such as age-, gender- and work practices could be correlated with work-related pain.

In this context, a high risk of bias has to be taken into account because people with MSD are much more inclined to respond to a questionnaire related to MSD.

The musculoskeletal disorders in dentists can probably be attributed to long working hours in static positions mostly in incorrect work postures without long breaks as well as to recurrent and repetitive movements [6, 13, 17].

The aforementioned static positions can be defined as constrained postures, in which a physical exertion takes places through continuously taking a particular position or holding the limbs at a certain angle during this exertion. Static work procedures entailing holding something without any support causes additional muscular strain as muscle endurance is required for the performance of the respective tasks in isometric positions. Moreover, the lack of motion impedes the blood flow necessary for tissue recovery [18]. The longer or more frequently static strains occur, the greater the risk of injury due to overuse of muscles, joints and other tissues [18].

From an occupational health perspective, static positions are mainly associated with bending, kneeling or squatting relevant for craft activities. For dentists’ work, static positions are determined by the design and structure of the workstation and inventory with the corresponding unilateral body or angle positions [19, 20].

Static body postures are quite often observed in the performance of dentists’ activities in which permanently forced loads have an effect on the musculoskeletal system. Moreover, they limit and define the posture and movement capabilities of the practicing dentist. The scope of movement, respectively the movement versatility, is low whereby a prolonged contraction, in particular of the trunk musculature with lower to medium load through the body weight, can be noted. [21]. Among dentists, many of the constrained postures in awkward angle value positions, in particular in the head-trunk area, can further be observed. These postures partly need to be maintained for a longer period of time during the treatment of a patient. A previously performed kinematic analysis of dentists with the same parameters that are used in this study has already shown that there are typical posture that are taken during tasks essential to the dental treatment of patients. The postures in the area of the cervical and thoracic spine have higher angular values during treatment compared to other dental tasks [22].

In occupational science, a static body posture is defined as a posture that is being held for more than four sec [23, 24]. Primarily, the musculature operates isometrically and not eccentrically or concentrically. Other significant factors are the frequency of occurrence, the breaks during movements and the duration (temporal component) for which a static body posture is held.

To gain insights into static body postures among dentists, a certain number of studies have already employed the valid and reliable RULA method (rapid upper limb assessment) [25]. This measures the neck, trunk and upper limbs postures along with muscle function and external loads as well as registering static positions lasting longer than one min [25]. This method was designed to record the work hazards associated with those risk factors regarding work-related upper limb disorders. A coding system to compile a list of activities shows the extent to which intervention is necessary to reduce the risk of injuries caused by physical strains of the worker [25].

Golcha et al. [26] conducted an investigation to detect ergonomic risk factors and their association with musculoskeletal disorders among 104 Indian dentists by using the RULA method. The authors discovered that the average body posture of the subjects was characterized by neck flexion and neck twisting, shoulder abduction and excessive forward trunk bending. They further pointed to the connection between the action level and the increasing frequency with regard to the reported health disorders in the back, neck and shoulder region. They thus concluded that RULA is a suitable method for evaluating body postures among dentists. A further analysis productively employed the RULA method to compare the body postures during simulated treatment of 60 dentistry students at two different dentist chairs with regard to musculoskeletal disorders [18]. They conclude that using a “Bambach Saddle Seat” maintains an acceptable working posture and reduces work-related musculoskeletal disorders. Although Golcha et al. [26] do not mention protective postures. Park et al. [27] also examined the work postures of three dentists with the RULA method. They concluded that the selected treatment positions need to be assessed as “improvement required,” respectively “instant improvement required” of these postures, whereby the risk of musculoskeletal disease is particularly high in the lower back and neck region. They further recommend that dentists should increase the number of breaks as well as execute muscle strengthening exercises to prevent diseases.

Based on the findings gained from the application of this screening method by observation (RULA), a kinematic posture analysis (CUELA) is intended to give detailed insights into the constrained postures of the dental work day.

The kinematic analysis with the CUELA system has already been used successfully in several studies [28–30], including dentists and orthodontists [22, 31]. So far, however, only the joint angles of the performed activities have been analyzed in comparison to ergonomic layouts. A separate kinematic analysis of static body positions has not yet taken place. The results already known are due to the observation method by using RULA [18, 26, 27], which is observational-dependent, but has nevertheless proven itself. The continuous metrological registration of postures and joint angles enables a more specific quantification of these unfavorable (non-neutral) postures. The kinematic analysis (CUELA) was combined with the observational method so that the conducted joint angles can be exactly assigned to the performed activity. This is based on a modified software based on the work of Mache et al. [32].

A kinematic analysis of dentists and orthodontists delineated the temporal positions during treatment and other activities in the dental workplace and classified the positions according to existing ergonomic standards [31]. Generally, both groups performed treatment activities in neutral or medium postures. However, dentists had slightly more unfavorable postures for a longer time of their work day. Similar results were observed by Ohlendorf et al. [22] in a separate analysis of dentists by using the same measurement system.

The objective of this approach is to provide insights into those dental, respectively non-dental, static activities during which these positions occur most frequently. These sets of kinematic data act as reference values that can be used for comparing new dental workplace conditions or can help to improve dental treatment chairs or the inventory arrangement. Such kinematic reference data of static positions does not exist for real medical situations as of yet.

To enhance the data analysis, the movement analysis will be combined with an objective activity analysis [32] and classified into the following the categories: (I) “treatment” (II) “office” and (III) “other activities.” Although the study population is the same as in the previously published study [22], the collected data has been subjected to a different analysis with different underlying hypotheses.

The following hypotheses are to be examined in this study:

### Hypothesis 1

: In treatment and office activities the total percentage of non-neutral static postures is higher in the trunk area than in the head and neck area.

### Hypothesis 2

: During treatment and office activities static postures are usually short (less than 30 s).

## Methods

### Subjects

In this study, 21 (11f/10 m) dentists, who work either as postgraduate training assistants or as dental professional were individually observed in his/her regular dental office (21 dental offices in total). Their average age was 40.1 ± 10.3 years, body height: 175.9 ± 8.2 m (160.0–190.0 m) and their average work experience was 10.6 ± 9.9 years. All subjects were right-handed and treated from the right side of the dental unit. The setup of the dental practice was almost comparable since all subjects sat on an ergonomic treatment chair (produced by different manufacturers). The height of each of the treatment units could also be adjusted.

According to their own statements, none of the participants showed signs of functional impairment or ailments related to the musculoskeletal system. Furthermore, previous injuries of the musculoskeletal system had to have occurred more than two years prior to the study. All participants were members of the Association of Dentists of Hessen (Germany). They were all registered in a publicly accessible register which is the source wherein all dentists in the Frankfurt/Main area can be contacted. The participants in this study were recruited from this list.

This study was approved by the Ethics Committee (135/14) of the Goethe University in Frankfurt am Main. All participants signed an informed consent in advance.

### Measuring system: CUELA

The posture analysis was conducted by using the CUELA system (computer-assisted acquisition and long-term analysis of musculoskeletal loads) which was developed at the Institute for Occupational Safety and Health of the German Social Accident Insurance (IFA; Sankt Augustin/Germany) [33, 34]. The system includes sensors (accelerometers [ADXL 103/203] and gyroscopes [muRata ENC-03R] for the head, arms, legs, back, potentiometers [Contelect] for back torsion). This results in a kinematic movements reconstruction. The sensors on the arms and legs as well as in the thoracic and lumbar spine areas were attached under the clothing. To measure head movements, the study participant wears a headband with sewed-on sensors [35].

The CUELA system measured with a sampling frequency of 50 Hz and an angular resolution of approximately ±1°. The measured angular joints accurately represent the dynamic movements as illustrated in [22].

### Measuring system: Objective activity analysis with mini-PC

The computer program, developed especially for activity analysis, records the workflows of the dentists in real time on a portable hand-held computer (UMPC, Samsung Q1, Samsung Electronics GmbH, Schwalbach, Germany) on a second-by-second basis. The computer program covers the full range of possible activity categories for a detailed description of the work spectrum of a dentist. In this way, the actual activity could be designated as such, and the duration of this activity could be determined within the working day. For a more detailed description of the system, see the method paper by Mache et al. [32, 36].

### Experimental procedure

As described previously [22], prior to the experiment, the work behavior of dentists was documented through precise observations and analyses. The results were discussed with dentists themselves as well. The respective tasks were subsequently implemented into the activity analysis software. The range of dentist activities was divided into the following three categories: (I) “treatment,” (II) “office” and (III) “other activities.” These categories comprised 18 activities; each activity described exactly one of the many tasks involved in the day-to-day work of a dentist.

For each participant, an average work day of a dentist was randomly selected for measuring time. Prior to the commencement of the workday and within the scope of the kinematic analysis, all sensors of the CUELA system were attached to the participants’ arms, legs and head as well as on the spine. Parallel to the recording through the CUELA system, observers supported the participants and documented every movement taken by the dentist by means of task analysis on the hand-held computer.

### Evaluation

The CUELA software (IFA; Sankt Augustin, Germany) allows for a temporal allocation of the motion patterns found in the individual activities of dentists by synchronizing the activity analysis with the CUELA measurement. In the context of the evaluation, activities were preselected based on their relevance and the percentage of the duration of the treatment of patients.

The angle values of each body region (evaluation parameters) are assigned to a color-coded angle range in accordance with ergonomic standards by means of a traffic light system (red/yellow/green). Based on the respective colors, body postures are assessed as awkward, moderate (partly acceptable) or neutral [37–39]. Corresponding to these classification criteria, the percentage share of each evaluation parameter for each activity (category I, II and III) is calculated and assessed as to whether it has been conducted in neutral, moderate or awkward body posture. In addition, the percentage shares of the moderate and awkward body positions are added and summarized as non-neutral body postures.

On this basis, the proportional static share of each evaluation parameter for the respective task is delineated as a further evaluation component. This share of stasis (termed: *total percentage of stasis*) refers to static postures, which are assessed according to ergonomic standards as moderate or awkward and which are, following the ISO norm [23], maintained for more than four s. Accordingly, neutral postures will not be taken into account. Within the scope of this study, it can further be assumed that body postures can be maintained significantly longer than four seconds.

The RULA method (rapid upper limb assessment) [25], which has already been applied for measuring postures of dentists, examined within the framework of a posture-related screening method for static positions which last longer than one min. This duration, however, has not been further specified.

Following these valuation methods, in this study in addition to the dividing line of ≥4 s, a further differentiation between body postures which last longer than 60 s, between 30 and 60 s, between 10 and 30 s and between 4 and 10 s will be made (termed*: stasis components*).

Furthermore, the ratio of the total percentage stasis share and the percentage share of the total non-neutral postures is calculated. This ratio indicates the extent of the static percentage of the posture within the non-neutral postures (termed: *total percentage of stasis of non-neutral postures*).

## Results

The measurements generated 108.6 h (6986.4 min) of usable data material, minus the non-related activities (such as breaks or going to the toilet). In total, 43% (2785 min) of the total time is subsumed in category I “treatment,” 24% (1544 min) in the category II “office” and 33% (2187 min) in category III “other activities.”

The category “treatment” encompasses ten activities, with the three most important tasks with the longest duration “craft activities” and “screening” taking up 90% (2481 min) of the total treatment time. In “office” (II), 90% (1385 min) of the working time is taken up by entering data into files, respectively computer work as well as all kinds of desk work, such as consulting files and findings as well as planning models. In the category “other activities” (III), the task “conversation” (73%, 1606 min) occupies the largest percentage of time (Table 1, Table 2).

**Table 1:** Static percentages of the performed body postures ≥4 s during treatment

Evaluation parameter [°]	Activity	Duration [min]	Stasis 4–10s [%]	Stasis 10–30s [%]	Stasis 30–60s [%]	Stasis ≥60s [%]	Total percentage of stasis [%]	Percentage of non-neutral postures [%]	Total percentage of stasis of non-neutral postures [%]
Head tilted to the front (HT_f)	Impression	40.48	5.8	1.5	----	----	7.3	48.2	14.5
	Extracting a tooth	63.83	7.3	2.8	----	----	10.1	85.3	11.6
	**Handicraft activities**	**1546.47**	**12.7**	**6**	**0.5**	**----**	**19.2**	**67.9**	**27.4**
	Conservatory dentistry	8.89	11.5	10.7	6.2	----	28.4	66.5	36.1
	Breaks during treatment	51.15	3.5	----	----	----	3.5	35.5	5.9
	X-ray	26.27	1.3	----	----	----	1.3	40.6	2.4
	Pain diagnostic	23.73	11.9	----	----	----	11.9	65.9	16.6
	Syringe	89.55	17	26.1	5.3	----	48.5	78	58.4
	**Screening**	**249.07**	**11**	**1.8**	**0.2**	**----**	**13**	**67.1**	**16.5**
	**Contra-angle/ultrasonic handpiece**	**685.52**	**22.7**	**14.5**	**1.1**	**0.2**	**38.5**	**79.9**	**46**
Head tilted to the right (HT_r)	Impression	40.48	1.9	----	----	----	1.9	26.5	4.4
	Extracting a tooth	63.83	4.5	0.7	----	----	5.2	75.7	6.9
	Handicraft activities	1546.47	8.1	3.3	----	----	11.5	50.4	20.8
	Conservatory dentistry	8.89	13.6	10.2	7.1	----	30.9	60.9	39.8
	Breaks during treatment	51.15	1.8	1.6	----	----	3.4	22	7.6
	X-ray	26.27	2.3		----	----	2.3	34.7	7.1
	Pain diagnostic	23.73	8.6	1.5	----	----	10.1	58.2	16.9
	Syringe	89.55	14.2	23.9	4.7	----	42.7	69.6	56.2
	Screening	249.07	7.2	0.7	----	----	7.9	56.7	12.8
	**Contra-angle/ultrasonic handpiece**	**685.52**	**16.7**	**8.1**	**0.3**	**----**	**25.2**	**62.7**	**35.5**
Neck curvature to the front (NC_f)	Impression	40.48	3.8	0.5	----	----	4.3	27.9	10.7
	Extracting a tooth	63.83	6.2	2.5	----	----	8.6	46.5	16.6
	Handicraft activities	1546.47	8.6	4.1	0.2	----	12.9	42.8	23.6
	Conservatory dentistry	8.89	0.9	----	----	----	0.9	25.5	4.7
	Breaks during treatment	51.15	5.9	3.1	----	----	9	41.3	11.9
	X-ray	26.27	1.2	2.2	----	----	3.4	36.7	5.1
	Pain diagnostic	23.73	10.8	1.2	----	----	12	45.6	19.9
	Syringe	89.55	8.4	12.8	2.6	----	23.8	40.8	33
	Screening	249.07	8.1	2.6	0.2	----	10.9	43.1	17.7
	Contra-angle/ultrasonic handpiece	685.52	12.5	10	0.9	----	23.3	44.8	36.1
Neck curvature to the right (NC_r)	Impression	40.48	2.6	----	----	----	2.6	24.5	8.2
	Extracting a tooth	63.83	4.4	1.8	----	----	6.2	61.6	8.4
	Handicraft activities	1546.47	6.6	2.8	----	----	9.4	42.4	17.9
	Conservatory dentistry	8.89	7	4.8	7.1	----	18.8	49.9	32.4
	Breaks during treatment	51.15	2.9	1.6	----	----	4.5	26	13.8
	X-ray	26.27	2.7	----	----	----	2.7	38.1	7.3
	Pain diagnostic	23.73	7.5	0.7		----	8.2	43.5	16.7
	Syringe	89.55	10.1	14.9	2.8	----	27.8	47.5	37.2
	Screening	249.07	6.9	0.8	----	----	7.8	44	12.9
	Contra-angle/ultrasonic handpiece	685.52	13.9	7.4	0.6	0.2	22	52.5	32.3
TS inclination to the front (TSI_f)	Impression	40.48	4.9	1.1	----	----	6	15.9	24.4
	Extracting a tooth	63.83	16.5	16.2	2.8	----	35.5	72.9	46.9
	Handicraft activities	1546.47	11.1	10.3	1.3	0.2	22.9	39.6	44
	Conservatory dentistry	8.89	7.6	8.9	14.6	----	31	42.1	56.8
	Breaks during treatment	51.15	4	4.6	----	----	8.6	16.2	21.4
	X-ray	26.27	7.9	2	----	----	9.9	36.7	23.3
	Paindiagnostic	23.73	13.9	10.1	----	----	24	42.7	37.1
	Syringe	89.55	6.2	24.7	10.7	1.2	42.9	52.3	53.8
	Screening	249.07	13.8	8.1	0.5		22.4	40.4	37.9
	**Contra-angle/ultrasonic handpiece**	**685.52**	**15.2**	**22.4**	**6.4**	**0.4**	**44.3**	**60.4**	**61.7**
TS inclination to the right (TSI_r)	Impression	40.48	3.7	0.6	----	----	4.3	12.6	10.1
	Extracting a tooth	63.83	5.4	6.3	0.9	----	12.6	22.5	35.5
	Handicraft activities	1546.47	3.6	3.7	0.9	0.2	8.2	16.1	25.3
	Conservatory dentistry	8.89	13.7	20.4	14.9	----	49	68.1	53.2
	Breaks during treatment	51.15	2.1	1.4	----	----	3.5	11.6	10.5
	X-ray	26.27	2.6	3.3	----	----	5.8	22.6	14
	Pain diagnostic	23.73	2.3	5.2	2.6	----	10	19.2	20.3
	Syringe	89.55	2.1	15.1	9.7	1.7	28.7	33.9	38.2
	Screening	249.07	10	6.5	0.5	----	17	32	28.9
	Contra-angle/ultrasonic handpiece	685.52	4.5	9	2.2	0.4	16.2	23.1	29.8
	Impression	40.48	3.8	0.5	----	3.8	4.3	27.9	10.7
LS inclination to the front (LSI_f)	Extracting a tooth	63.83	6.2	2.5	----	6.2	8.6	46.5	16.6
	Handicraft activities	1546.47	8.6	4.1	0.2	8.6	12.9	42.8	23.6
	Conservatory dentistry	8.89	0.9	----	----	0.9	0.9	25.5	4.7
	Breaks during treatment	51.15	5.9	3.1	----	5.9	9	41.3	11.9
	X-ray	26.27	1.2	2.2	----	1.2	3.4	36.7	5.1
	Pain diagnostic	23.73	10.8	1.2	----	10.8	12	45.6	19.9
	Syringe	89.55	8.4	12.8	2.6	8.4	23.8	40.8	33
	Screening	249.07	8.1	2.6	0.2	8.1	10.9	43.1	17.7
	Contra-angle/ultrasonic handpiece	685.52	12.5	10	0.9	12.5	23.3	44.8	36.1
Back curvature to the front (BC_f)	Impression	40.48	2.7	0.6	----	----	3.2	6	8.3
	Extracting a tooth	63.83	8.1	13.7	2	----	23.8	34.9	43.3
	Handicraft activities	1546.47	1.8	2.1	0.3	0.2	4.3	6.6	14.1
	Conservatory dentistry	8.89	1.8	----	----	----	1.8	2.2	1.9
	Breaks during treatment	51.15	1	0.8	----	----	1.8	2.7	14.6
	X-ray	26.27	7.1	2.3	----	----	9.4	19.4	30.8
	Pain diagnostic	23.73	4.1	4.4	----	----	8.5	9.7	12.8
	Syringe	89.55	1.3	6.6	6.9	1.1	16	18.1	20.3
	Screening	249.07	1.6	1.7	0.2	----	3.6	5.3	8.1
	Contra-angle/ultrasonic handpiece	685.52	1.8	4.7	1.1	0.3	7.9	9.5	16
Back curvature to the right (BC_r)	Impression	40.48	2	2	0.7	----	2.7	7.9	10.3
	Extracting a tooth	63.83	3	3	2.3	0.8	6.1	10.5	26
	Handicraft activities	1546.47	2	2	2.4	0.4	5	9.2	21.4
	Conservatory dentistry	8.89	5.6	5.6	19.9	7.4	32.9	40.1	57.3
	Breaks during treatment	51.15	1.3	1.3	----	1.1	2.4	5.7	4.9
	X-ray	26.27	1.9	1.9	1.5	----	3.4	15.2	8.8
	Pain diagnostic	23.73	0.9	0.9	1.6	----	2.5	6.8	7.3
	Syringe	89.55	0.9	0.9	6.4	5.1	16.1	20	21.6
	Screening	249.07	6.3	6.3	6.5	0.2	13.1	20.2	28.2
	Contra-angle/ultrasonic handpiece	685.52	2.2	2.2	5.5	1.4	9.5	13.1	21.4
Back torsion to the right (BT_r)	Impression	40.48	7	5.8	1.3	----	14.2	25	30.5
	Extracting a tooth	63.83	8.3	8.5	1.9	----	18.6	32.9	34.5
	Handicraft activities	1546.47	7.2	9.2	2.6	0.5	19.4	32.6	36.3
	Conservatory dentistry	8.89	3.6	----	----	----	3.6	12.4	4.9
	Breaks during treatment	51.15	4	11.1	4.3	2.4	21.8	35.3	26.7
	X-ray	26.27	8.3	5.5	2.7	----	16.6	45.3	22.8
	Pain diagnostic	23.73	3.6	11.6	----	----	15.3	25.6	27.8
	Syringe	89.55	2	12.8	9	2.6	26.4	34.4	34.4
	**Screening**	**249.07**	**8.2**	**14.3**	**2.6**	**2.8**	**27.9**	**40.8**	**42.9**
	**Contra-angle/ultrasonic handpiece**	**685.52**	**5.1**	**15.5**	**7.7**	**2.3**	**30.7**	**39.7**	**42.1**

Results to be emphasized are marked in bold. Key: Total percentage of statics = sum of all static postures in the moderate and unfavorable ergonomic range during all activities

**Table 2:** Static percentages of the performed body postures ≥4 s during office work and other activities

Evaluation parameter [°]	Activity	Duration [min]	Stasis 4–10s [%]	Stasis 10–30s [%]	Stasis 30–60s [%]	Stasis ≥60s [%]	Total percentage of stasis [%]	Percentage of non-neutral postures [%]	Total percentage of stasis of non-neutral postures [%]
Head tilted to the front (HT_f)	Reading patient files	420.77	5	2.2	0.3	----	7.4	36.8	14.3
	Office work	963.92	4.7	2.8	0.3	0.4	8.2	31.6	16.6
	Phone calls	159.20	3.7	2.3	0.3	----	6.3	20.3	18.3
	Conversation	1605.79	1.1	0.2	----	----	1.3	25.5	3.3
	Hygiene	75.98	0.4	----	----	----	0.4	35.3	0.5
	Take/deposit instruments	184.52	2.9	0.5	----	----	3.3	57.9	4.4
	Laboratory	178.35	11.6	6.7	----	----	18.2	54.5	24.3
	Walk	142.08	0.1	----	----	----	0.1	27.8	0.2
Head tilted to the right (HT_r)	Reading patient files	420.77	2.3	0.4	0.3	----	3	21.9	6.2
	Office work	963.92	3.2	1.2	0.1	0.3	4.9	21.8	15.1
	Phone calls	159.20	2.1	0.8	----	----	2.9	15.7	9.6
	Conversation	1605.79	1	0.3	----	----	1.3	19.8	3.7
	Hygiene	75.98	0.5	0.3	----	----	0.7	20.9	1.5
	Take/deposit instruments	184.52	1.7	0.1	----	----	1.8	34.3	3.6
	Laboratory	178.35	6.1	4.7	1.1	----	12	36.6	23.2
	Walk	142.08	0.2	----	----	----	0.2	22.7	0.2
Neck curvature to the front (NC_f)	Reading patient files	420.77	8.5	5.5	1.3	----	15.4	52.9	20
	Office work	963.92	8.5	7.3	2.6	0.6	19	54	29.7
	Phone calls	159.20	8	8.2	0.3	----	16.5	38.7	29.2
	Conversation	1605.79	2.6	0.6	----	----	3.2	44.9	5
	Hygiene	75.98	0.5	----	----	----	0.5	31.6	0.6
	Take/deposit instruments	184.52	2.6	0.5	----	----	3.1	43.3	5.2
	Laboratory	178.35	7	5	1.1	----	13	44.3	27.1
	Walk	142.08	0.3	0.1	----	----	0.5	33.7	1
Neck curvature to the right (NC_r)	Reading patient files	420.77	2.9	1.4	0.4	----	4.7	24.9	10.8
	Office work	963.92	3	1.3	----	0.5	4.7	21.1	16.1
	Phone calls	159.20	2.6	1.8	----	----	4.4	20	10.8
	Conversation	1605.79	1.3	0.3	----	----	1.6	19.9	5.1
	Hygiene	75.98	0.1	0.3	----	----	0.4	20.6	0.4
	Take/deposit instruments	184.52	1.4	0.3	----	----	1.7	34.5	3.3
	Laboratory	178.35	5.1	4	1.1	----	10.3	34.3	24.9
	Walk	142.08	0.3	----	----	----	0.3	23.6	0.4
TS inclination to the front (TSI_f)	Reading patient files	420.77	7.2	8.5	2.6	1.5	19.9	35.3	32.8
	Office work	963.92	6	10.1	5	3.1	24.3	35	39.8
	Phone calls	159.20	2.9	3.2	3	2.2	11.3	16.2	27.4
	Conversation	1605.79	3	2.7	0.6	0.2	6.5	17.8	13.4
	Hygiene	75.98	1	----	----	----	1	18.2	1.8
	Take/deposit instruments	184.52	6.5	2.6	0.4	----	9.5	49.8	12.2
	Laboratory	178.35	2.6	7.7	9.6	13.5	33.5	42.2	54
	Walk	142.08	0.3	0.1	----	----	0.4	10	0.6
TS inclination to the right (TSI_r)	Reading patient files	420.77	2.2	2.5	1.1	----	5.8	12.1	12
	Office work	963.92	1.3	2.6	2.6	4.5	11	14.2	26.8
	Phone calls	159.20	0.9	2.7	0.8	0.6	5.1	8.6	14.1
	Conversation	1605.79	1.2	1.1	0.5	0.2	3	11.4	8.4
	Hygiene	75.98	1.1	----	----	----	1.1	11.9	2
	Take/deposit instruments	184.52	1.2	0.1	----	----	1.3	13.3	2.5
	Laboratory	178.35	0.9	1.2	0.4	0.7	3.2	8.5	13.3
	Walk	142.08	0.3	0.3	----	----	0.6	16.1	1
LS inclination to the front (LSI_f)	**Reading patient files**	**420.77**	**12.2**	**13.2**	**4.4**	**2.7**	**32.5**	**60.3**	**39.9**
	**Office work**	**963.92**	**13.8**	**24.1**	**10.4**	**5.7**	**54**	**78.2**	**64**
	Phone calls	159.20	13	16.6	11.3	8.3	49.1	73.6	58.6
	Conversation	1605.79	8.4	5.9	1.2	0.7	16.1	55.1	22.2
	Hygiene	75.98	2.6	----	----	----	2.6	45.1	3.9
	**Take/deposit instruments**	**184.52**	**9.3**	**3.6**	**0.7**	**----**	**13.6**	**63.7**	**16.5**
	Laboratory	178.35	6.3	11.4	10.8	13.5	42	59.8	50.9
	Walk	142.08	0.2	0.1			0.3	31.4	0.5
Back curvature to the front (BC_f)	Reading patient files	420.77	2.8	5.3	1.6	1	10.7	15.2	18.6
	Office work	963.92	1.2	1.9	0.9	0.1	4.1	6.9	8.5
	Phone calls	159.20	0.4	0.4	0.4	----	1.3	3	11.5
	Conversation	1605.79	0.5	0.4	----	----	0.9	3.3	3.1
	Hygiene	75.98	0.7	----	----	----	0.7	7.3	1.2
	Take/deposit instruments	184.52	5	2.6	----	----	7.6	27.6	12.1
	Laboratory	178.35	0.5	0.1	----	0.6	1.2	3.9	7.6
	Walk	142.08	0.1	----	----	----	0.1	4.6	0.2
Back curvature to the right (BC_r)	Reading patient files	420.77	1.8	2.1	1.2	----	5.1	9	11.7
	Office work	963.92	0.6	1.3	1.2	5.4	8.5	10.3	25
	Phone calls	159.20	0.3	0.7	0.3	0.6	2	4	6.3
	Conversation	1605.79	0.7	0.6	0.3	0.3	2	7	6.7
	Hygiene	75.98	0.9	----	----	----	0.9	7.6	1.7
	Take/deposit instruments	184.52	1	0.1	----	----	1.1	10.2	2.6
	Laboratory	178.35	0.6	1.6	0.6	2.5	5.4	8.4	20.1
	Walk	142.08	0.1	0.1			0.3	10.6	0.6
Back torsion to the right (BT_r)	Reading patient files	420.77	7.4	9.7	5.2	2.1	24.4	39.7	35.6
	**Office work**	**963.92**	**4.2**	**10.7**	**5.7**	**6.1**	**26.8**	**34.7**	**51.9**
	Phone calls	159.20	3	7.5	6	5.2	21.8	28.2	38.3
	Conversation	1605.79	5.4	5.9	2.4	0.9	14.6	32.6	23.4
	Hygiene	75.98	1.8	0.6	----	----	2.4	31.1	3.4
	Take/deposit instruments	184.52	5.5	2.5	0.4	----	8.4	34.1	12.7
	Laboratory	178.35	3.7	4.4	5.1	3.5	16.7	27.4	40.7
	Walk	142.08	0.2	0.3	----	----	0.5	28.9	0.7

Results to be emphasized are marked in bold. Key: Total percentage of stasis = sum of all static postures in the moderate and unfavorable ergonomic range during all activities

This result section will primarily take those activities into account which are most frequently performed. The following description of this data focuses on those non-neutral body positions whose percentage share is ≥60%. The threshold of significant results regarding the total percentage of stasis of non-neutral postures is 40% (marked in bold).

### Category I: Treatment

Table 1 contains the percentage share of activities of moderate and awkward postures which are summed up and described as non-neutral body postures. Further, Table 1 contains the percentage specifications of the static shares of the performed postures of the total percentage of stasis, their temporal differentiation, as well as the total percentage of stasis of non-neutral postures.

In the *head and neck area*, for the head tilted to the front (HT_f) during “craft activities” the percentage share of non-neutral postures is 67.9%, from which 27.4% were conducted statically. Within the distribution of static postures, the stasis percentage of postures between 4 and 10 s is the highest at 12%, followed by postures between 10 and 30 s at 6%. During the activity “contra-angle/ultrasonic handpiece,” 79.9% of the postures are in the non-neutral range with a total percentage of stasis of 46%. With regard to the static postures (38.5%), 22.7% were held for 4–10 s and 14.5% for 10–30 s.

In the category “screening,” the percentage share of non-neutral postures is at 67.1% with a total percentage of stasis of 16.5%, whereof 11% of the static postures are executed for up to 10 s.

Conclusive data for the head tilted to the right (HT_r) can only be registered during the activity “contra-angle/ultrasonic handpiece.” The percentage share of non-neutral activities is 62.7% with a total percentage of stasis of 35.5%. At 66.3%, the largest proportion of static postures is conducted between 4 and 10 s.

In the *trunk area*, the TS inclination to the front (TSI_f) for the same activity shows a large percentage share of non-neutral postures with 60.4%, whereof 61.7% are performed in a static position. The duration of the static postures lies predominantly between 4 and 10 s (34.3%) and 10–30 s (36.3%).

The final significant movement is the back torsion to the right (BT_r) during the activities “screening” and “contra-angle/ultrasonic handpiece.” During both tasks, the total percentage of stasis within the non-neutral postures is approximately equal (42.9%, respectively 42.1%). In this case again, with regard to the relative stasis specification, the duration of static postures between 4 and 10 s (29.4%, respectively 16.6%) and between 10 and 30 s (33.3% respectively 36.8%) account for the largest share.

### Categories II and III: Office work and other activities

Table 2 comprises the percentage specification of the static share of the performed postures of the total percentage of stasis, their temporal differentiation as well as the total percentage of stasis of non-neutral postures for the tasks “office” and “other activities.”

For the category II (“office”), the values of the *head-neck-area* have a percentage of non-neutral postures of <60% and a total percentage of stasis of non-neutral postures of <40%.

In the *trunk area,* the LS inclination to the front (LSI_f) during the tasks “reading patient files” and “office work” accounts for 60.3% and 78.2%, respectively, of the total of non-neutral postures. During the first activity, in conformity with the stasis components, these are the most frequently performed with 13.2% between 10 and 30 s, followed by 12.2% between 4 and 10 s. In “office work,” most tasks last between 10 and 30 s at 24.1% and between 4 and 10 s at 13.3%. In addition, 10.4% of the activities last between 30 and 60 s.

Moreover, a strikingly high total percentage of stasis of non-neutral postures (51.9%) can be observed during “office work” for the back torsion to the right (BT_r). Here, the share of non-neutral postures amounts to 34.7%. Thereby, 10.7% of the static activities are performed for 10 to 30 s. With a percentage share of 6.1% in this segment, this activity was conducted statically for more than 60 s.

In category III (“other activities”), in the *head-neck area*, the share of non-neutral postures amounts to <60% and the total percentage of stasis of non-neutral postures to <40%.

Merely the LS inclination to the front (LSI_f) is noticeable during the task “take/deposit instruments” as the share of non-neutral postures is 63.7%. The total percentage of stasis of non-neutral postures amounts to16.5%. At 9.3%, these static activities are performed for 4 to 10 s.

## Discussion

Dental work processes are for the most part predetermined and these circumstances result in awkward body postures, which then require the dentist to work in constrained postures. These awkward postures result in static overexertion of the musculature which can lead to musculoskeletal disorders. Accordingly, many dental tasks present a potential hazard to the musculoskeletal system, such as prolonged bending or twisting of the trunk or sitting in a predefined uninterrupted fixed posture.

Our previous results showed that depending on the activity, unfavorable postures were observed, especially in the head-and-neck area in accordance to ergonomic layouts. In comparison to office work (II), the greater angle values of head and cervical spine area showed that treatment activities were increasingly conducted in forced postures (dentist and orthodontist) [22, 31]. Therefore, this study separately analyzes static positions and, in particular, those static postures that were held for at least four seconds during the activities in a dental work day by means of a kinematic analysis based on previous results.

For the analyzed activities, the total percentage of stasis of non-neutral postures during “treatment” (I) is higher in the head area than in the trunk area, whereby the TS inclination to the front (TSI_f) and the back torsion to the right (BT_r) during the activity “contra-angle/ultrasonic handpiece” as well as latter movement during “screening” are three exceptions. In contrast, in the categories II and III, the total percentage of stasis of non-neutral postures is higher in the trunk area than in the head area. It can, therefore, be concluded that with regard to the execution there is a difference between category I and II, as well as I and III, in terms of the required static holding of the muscles. In the head-trunk area, during “treatment” most static body postures are held for 4–10 s, followed by postures lasting up to 30 s. Based on the share of non-neutral postures or the total percentage of stasis of non-neutral postures, it becomes apparent that the most frequently held static posture is the entire upper body (head and trunk area) when it is anteriorly tilted while the back is inclined to the right. During the activity “contra-angle/ultrasonic handpiece” the head is simultaneously inclined to the left. The fact that the lower back and neck are predominantly subject to the risk of musculoskeletal disorders has been discovered by means of the RULA method by Park et al. [27]. These findings can be confirmed by the results of the present kinematic data on static postures. Therefore, hypothesis 1 and 2 are verified.

Also in the analyzed activities of categories II and III, the share of static postures in the trunk area lies predominantly between 30 and 60 s. The static muscle contractions in the head area can be neglected here due to the fact that either the share of non-neutral postures or the total percentage of stasis of non-neutral postures was too low.

During “office work” (category II), however, the back was curved to the front for more than 30 s and even twisted to the right for a duration of 60 s at 6,1% (58,8 min of 693.9 min).

The categorical comparison clarifies that in category I (“treatment”) static muscle contraction in the entire upper body and head are required in order to perform dental tasks. In contrast, the other two categories comprise static postures predominantly in the trunk area.

The potential health hazards of the prolonged inclined postures during office work are thus not assessed as high as desk work because it occurs predominantly in supported postures and are accordingly not accompanied by static muscle strain. The tasks performed by the dentist during treatment, in contrast, have to be performed in an unsupported position and are thus connected to higher muscle strain. The stasis statistics of office work, which is mainly executed in supported positions, can therefore not be directly compared to the statistics of dental tasks [40, 41].

Our kinematic data can be used to define reference values for creating new and innovative dental workplace conditions, improving dentists’ treatment chairs or analyzing the inventory arrangement of the basic arrangements (four current existing possibilities). Such kinematic reference data does not exist for real medical situations. Previous studies have described posture patterns in dentistry employing the RULA method [25–27]. However, the results of these studies are less objective, reliable and valid than the kinematic CUELA data. In addition, the results of our study are quantitative.

The assessment of constrained postures invariably has to take into account the total duration of the corresponding activity as well as the respective percentage share of the singular stasis component. Consistently, only activities with relevant duration and significance for the dental work process were analyzed.

Another fact that has to be taken into account is the analysis of the head, thoracic and lumbar region of the upper body when these regions are not considered in relation to each other. At the moment, this extended analysis is not possible within the scope of the evaluation software.

Furthermore, the question, whether static awkward postures occur in all regions at the same time while executing one particular movement cannot be answered based on the current state of the evaluation software. A modification of the program could help to analyze this relationship in further investigations.

The evaluation procedure of the kinematic analysis measures the total duration and frequency of statically held postures. Although movement breaks in-between the singular tasks cannot be measured within the scope of this study, still it has to be considered that such breaks in-between working would relieve the strain on the spine. Dentists who have a well-trained muscular system can use these short breaks to recover and are therefore pain-free. This holds true for the dentists who have been recruited for this study. The postural habits, as well as the constitutional musculoskeletal state of such pain-free dentists, appear to be effective and preventive against work-related musculoskeletal disorders WRMSD. Such dentists, whose musculoskeletal system is not appropriately trained to adequately absorb such static effects, can be susceptible to WRMSD or perhaps already suffer from these disorders.

It is, moreover, not possible to differentiate between supported and unsupported postures by means of the present data. This would nonetheless be desirable as it would be possible to detect corresponding mechanics of impairment related to singular activities. Both aspects should be taken into account in further studies as the frequency and the duration of the breaks between different, respectively repetitive, tasks just like the differentiation between supported and unsupported activities are determining factors for the analysis of constrained postures. Up to now, there has not been any kinematic analysis of fine motor movements of fingers, hands, or wrist joints published (of those suffering from pain in the hands 25%,complain of pain in the fingers and hands and 44% in the wrist joints) has been published, even though precise and delicate tasks are necessary for dental work [2, 42]. The prevalence of these physical ailments is well-known. Moreover, the respective sensors are not integrated into the measuring system. As a result, it is difficult to implement them in field studies.

These work-related tasks are subject to predefined motion sequences which can result in work restriction, or transfer to another job, which in turn can lead to WRMSD. In addition to dentists, the high prevalence of MSDs in the head-neck region, respectively back region, can also be observed in other professions. These include professions that require predetermined motion sequences, such as physical therapists [43–45], hairdressers or [46–48] or beverage bottling workers [49]. For these occupations, only data from a postal questionnaire has been published to date. The WRMD of physiotherapist suffer most from hand or wrist pain and second most from lower back pain or neck and thoracic spine pain. Similar results have also been published for hairdressers and beverage bottling workers also using a questionnaire. Hassan et al. [48] even concluded that the high prevalence of WRMDs found in hairdressers highlights the importance of prevention symptoms by providing suitable furniture, equipment and work tools, and optimizing environmental conditions including the size of workplace and its organization. However, in these occupational professions with similar complaints to dentists no kinematic analysis has been carried out as of yet. A comparison based on the same technology (CUELA) would enable improvement of the working conditions for these occupations as well.

## Conclusion

In the course of a kinematic anamnesis, potential work hazards in dentistry regarding static postures as risk factors for musculoskeletal disorders can be registered that are associated with work-related tasks [31]. Manual activities (e.g. “craft activities,” “contra-angle” or “ultrasonic handpiece”) lead to constrained postures of dentists in non-neutral joint angles in treatment. As a result, the head position and the thoracic region remain in an anterior tilted, static position up to 30 s. In addition, the upper body is twisted. “Office work” or “reading patient files” resulted in a tilted forward and rotated static position in the lower back area (also being in non-neutral body positions). Dentists should be advised to change their seating position frequently in order to decrease static postures. Furthermore, the office workplace should be optimized to prevent rotations in this working position. Compensatory muscle strength and relaxation exercises for the musculoskeletal system, especially in the area of the cervical spine, could prevent these symptoms.

## Abbreviations

CUELA-System: Computer-assisted acquisition and long-term analysis of musculoskeletal loads
